# Supplementary material for: Sorting at embryonic boundaries requires high heterotypic interfacial tension
Source: Nat Commun. 2017 Jul 31;8:157. doi: 10.1038/s41467-017-00146-x (PMC5537356; doi:10.1038/s41467-017-00146-x)
Supplement: Supplementary file 2 — Supplementary Software 1 [file 41467_2017_146_MOESM2_ESM.zip › PottsModel/SrcPottsModel/doc/model/Lattice.PixelIterator.html]

Lattice.PixelIterator


JavaScript is disabled on your browser.


Skip navigation links


- Overview
- Package
- Class
- Use
- Tree
- Deprecated
- Index
- Help

- Prev Class
- Next Class

- Frames
- No Frames

- All Classes

- Summary:
- Nested |
- Field |
- Constr |
- Method

- Detail:
- Field |
- Constr |
- Method


model

## Class Lattice.PixelIterator

- java.lang.Object
- - model.Lattice.PixelIterator

- All Implemented Interfaces:
  :   java.util.Iterator<Pixel>

  Enclosing class:
  :   Lattice

  ---

    

  ```
  public class Lattice.PixelIterator
  extends java.lang.Object
  implements java.util.Iterator<Pixel>
  ```

- - ### Constructor Summary

    Constructors

    Constructor and Description

    `PixelIterator()`
  - ### Method Summary

    All Methods Instance Methods Concrete Methods

    | Modifier and Type | Method and Description |
    | --- | --- |
    | `boolean` | `hasNext()` |
    | `Pixel` | `next()` |
    | `void` | `remove()` |
    | `void` | `resetIterator()` |

    - ### Methods inherited from class java.lang.Object

      `equals, getClass, hashCode, notify, notifyAll, toString, wait, wait, wait`
    - ### Methods inherited from interface java.util.Iterator

      `forEachRemaining`

- - ### Constructor Detail


    - #### PixelIterator

      ```
      public PixelIterator()
      ```
  - ### Method Detail


    - #### hasNext

      ```
      public boolean hasNext()
      ```

      Specified by:
      :   `hasNext` in interface `java.util.Iterator<Pixel>`


    - #### next

      ```
      public Pixel next()
      ```

      Specified by:
      :   `next` in interface `java.util.Iterator<Pixel>`


    - #### resetIterator

      ```
      public void resetIterator()
      ```


    - #### remove

      ```
      public void remove()
      ```

      Specified by:
      :   `remove` in interface `java.util.Iterator<Pixel>`


Skip navigation links


- Overview
- Package
- Class
- Use
- Tree
- Deprecated
- Index
- Help

- Prev Class
- Next Class

- Frames
- No Frames

- All Classes

- Summary:
- Nested |
- Field |
- Constr |
- Method

- Detail:
- Field |
- Constr |
- Method
